# Characterizing the microbiota in gastrointestinal tract segments of *Rhabdophis subminiatus*: Dynamic changes and functional predictions

**DOI:** 10.1002/mbo3.789

**Published:** 2019-03-07

**Authors:** Wenjiao Tang, Guangxiang Zhu, Qian Shi, Shijun Yang, Tianyuan Ma, Shailendra Kumar Mishra, Anxiang Wen, Huailiang Xu, Qin Wang, Yanzhi Jiang, Jiayun Wu, Meng Xie, Yongfang Yao, Diyan Li

**Affiliations:** ^1^ College of Life Science Sichuan Agricultural University Ya’an China; ^2^ Farm Animal Genetic Resources Exploration and Innovation Key Laboratory of Sichuan Province Sichuan Agricultural University Chengdu China

**Keywords:** diets, gut microbiota, microbial function, Proteobacteria, *Rhabdophis subminiatus*

## Abstract

The gut microbiota helps the host to absorb nutrients and generate immune responses that can affect host behavior, development, reproduction, and overall health. However, in most of the previous studies, microbiota was sampled mainly using feces and intestinal contents from mammals but not from wild reptiles. Here, we described the bacterial profile from five different gastrointestinal tract (GIT) segments (esophagus, stomach, small intestine, large intestine, and cloaca) of three wild *Rhabdophis subminiatus* using 16S rRNA V4 hypervariable amplicon sequencing. Forty‐seven bacterial phyla were found in the entire GIT, of which Proteobacteria, Firmicutes, and Bacteroidetes were predominant. The results showed a significant difference in microbial diversity between the upper GIT segments (esophagus and stomach) and lower GIT segments (large intestine and cloaca). An obvious dynamic distribution of Fusobacteria and Bacteroidetes was observed, which mainly existed in the lower GIT segments. Conversely, the distribution of Tenericutes was mainly observed in the upper GIT. We also predicted the microbial functions in the different GIT segments, which showed that microbiota in each segments played an important role in higher membrane transport and carbohydrate and amino acid metabolism. Microbes in the small intestine were also mainly involved in disease‐related systems, while in the large intestine, they were associated with membrane transport and carbohydrate metabolism. This is the first study to investigate the distribution of the gut microbiota and to predict the microbial function in *R. subminiatus*. The composition of the gut microbiota certainly reflects the diet and the living environment of the host. Furthermore, these findings provide vital evidence for the diagnosis and treatment of gut diseases in snakes and offer a direction for a model of energy budget research.

## 1 INTRODUCTION

The gastrointestinal (GI) tract of most animals harbors hundreds of millions of microbes (Colston & Jackson, 2016). The vast majority of microbes that are closely linked to animal hosts are mainly present in the host's gut. The gut microbiota helps the host absorb nutrients and generate immune responses that can affect host behavior, development, reproduction, and overall health (Ellegaard & Engel, 2016; Ezenwa, Gerardo, Inouye, Medina, & Xavier, 2012; Lee & Hase, 2014). In addition, several factors play key roles in shaping the composition of an animal's gut microbiota, including diet, host phylogeny, gut morphology, and geographical environment (Ley, Lozupone, Hamady, Knight, & Gordon, 2008). Previous studies have shown that carnivorous, herbivorous, and omnivorous animals have different gut microbiota compositions (Ley, Hamady, et al., 2008; Xue et al., 2015). The gastrointestinal tract (GIT) of carnivorous animals is relatively simple compared to those of omnivores and herbivores (Schwab & Gänzle, 2011; Xue et al., 2015). It is widely believed that herbivores have the highest microbiome diversity followed by omnivores and then carnivores (Ley, Hamady, et al., 2008). At present, GIT microbiome studies have mainly focused on herbivores, omnivores, and domestic animals, whereas the gut microbial composition of carnivorous wild animals has yet to be widely explored. Using fecal samples to investigate the gut microbial community mainly reflects the composition of microbes in the lower GIT and does not reveal the differences in microbial composition between GIT segments or explore their functional roles (Suzuki & Nachman, 2016). Physiological variations in different GIT segments include chemical and nutrient gradients, both of which are known to influence bacterial community composition. Studies on the composition of microbiota in different GIT segments have been carried out on animals with various diets, including mice (Suzuki & Nachman, 2016), pigs (Zhao et al., 2015), bactrian camels (He et al., 2018), sheep (Zhang et al., 2018), alligators (Keenan, Engel, & Elsey, 2013), and snakes (Colston, Noonan, & Jackson, 2015).

Snakes (Squamata: Serpentes) are an important branch of amniotic ectothermic vertebrates that occupy every continent except Antarctica and nearly all biomes, including terrestrial, freshwater, and marine habitats (Zhao, 1998, 2006). They can reduce the quality and maintenance requirements of their gastrointestinal organs to reduce their standard metabolic rates for long periods of starvation and restore their ability to digest and absorb food immediately after ingesting prey (Starck & Beese, 2002). Snakes can be used as an ideal model organism for the study of animal energy budgets (Beaupre, 1996, 2002; Holmberg et al., 2002). Previous studies on microbiome variation among GIT segments have mainly concentrated on the rattlesnake and cottonmouth snake (Colston et al., 2015; Hill, Hanning, Beaupre, Ricke, & Slavik, 2008; Mclaughlin, Cochran, & Dowd, 2015). We investigated the gut microbiota composition and diversity in colubrid snakes, which could increase our knowledge of nutrient acquisition in the hidden lives of animals.

*Rhabdophis subminiatus* (Serpentes: Colubridae: Natricinae) is a medium‐sized natricine and falls under the least concern category by the International Union for the Conservation of Nature (IUCN) Red List. This snake is widely distributed in the southeastern region of China and South‐East Asia and is mostly found in rice fields and nearby water. *R. subminiatus* is diurnal and semi‐aquatic and feeds on amphibians and fish, but the diet is dominated by frogs and toads (Mohammadi & Hill, 2012; Zhao, 1998, 2006). *R. subminiatus* possesses nuchal glands, and it has been noted that they can store bufadienolides as well. Interestingly, bufadienolides are derived mainly from feeding on toads (Mohammadi & Hill, 2012). The bites of colubrid snakes can lead to severe envenomation, resulting in severe coagulopathy and transient hypertension (Nelwan, Adiwinata, Handayani, & Rinaldi, 2016). Moreover, the symptoms of such snake bites might be delayed for hours or days after the initial bite (Nelwan et al., 2016; Smeets, Melman, Hoffmann, & Mulder, 2010). The diet composition has been determined to have an impact on the host's gut microbiota (Kopečný, Mrázek, & Killer, 2010). Since the toad can affect the toxicity of the snake, is the intestinal microbial composition of the snake similar to that of the toad? The evolution of this interaction between prey and host requires a series of fundamental experiments to achieve a better understanding.

In this study, we used high‐throughput sequencing based on the Illumina HiSeq2500 platform to analyze the microbial community in the esophagus, stomach, small intestine, large intestine, and cloaca of *R. subminiatus*. Exploring the composition of microbiota and their potential function in different segments of the GIT could enhance our knowledge of ecology, host interaction, and adaptive evolution.

## 2 MATERIALS AND METHODS

### 2.1 Animals and sample collection

Three individual snakes were obtained from an area near a pond (Guangdong Province, China) on 13 October 2017. One subadult female (RS.1), one adult male (RS.2), and one adult female (RS.3) were collected; more detailed sample information is provided in Appendix A1. We examined the snakes while they fasted for 3 days in captivity prior to dissection. Then, snakes were euthanized with diethyl ether, and the abdomen was exposed by a sterile scalpel devoid of digesta in all digestive tracts. Fresh tissues were collected from different GIT segments, including the esophagus (ES.1, ES.2 and ES.3), stomach (ST.1, ST.2 and ST.3), small intestine (SI.1, SI.2 and SI.3), large intestine (LI.1, LI.2 and LI.3), and cloaca (C.1, C.2 and C.3). The tissues were then frozen in liquid nitrogen and stored at −80°C.

### 2.2 Genomic DNA extraction

Total genomic DNA from the gut tissues was extracted using a TIANamp Stool DNA Kit (Tiangen Biotech, Beijing) following the manufacturer's instructions with small modifications. We used 200 mg of each sample to study the microbes present on the mucosal surface and in the gut contents. The entire gut segment was immediately mechanically disrupted by sterile scissors in an EP tube containing 1.4 ml ASL (the frozen gut tissues were chopped into pieces with sterile scissors in an EP tube on an ice box, and the entire procedure was performed on a sterile test stand to prevent contamination). Then, blending was performed on the shaking table. The intestinal tissue was collected in the tube by brief centrifugation, the upper suspension (lysate) was taken for the extraction of microbial DNA, and the remaining pellet was discarded (this step was conducted to reduce the interference of host DNA during microbial DNA extraction). The EP tube was incubated at 70°C for 5 min. The next steps were carried out according to the manufacturer's instructions. Finally, the quality of DNA was determined by agarose gel electrophoresis, and the concentration was examined by a NanoDrop 3300 (Thermo Scientific, Chengdu).

### 2.3 PCR amplification and Illumina HiSeq platform sequencing

The V4 hypervariable regions of the 16S rRNA gene were amplified using the forward primer 515F (5′‐GTGCCAGCMGCCGCGGTAA‐3′) and the reverse primer 806R (5′‐GGACTACHVGGGTWTCTAAT‐3′) (Caporaso et al., 2012). All PCR was carried out in 30 μl reactions with 15 μl of Phusion High‐Fidelity PCR Master Mix (New England Biolabs), forward and reverse primers at 3 μl, approximately 10 μl of template DNA, and 2 μlddH_2_O. Thermal cycling consisted of initial denaturation at 98°C for 1 min, followed by 30 cycles of 98°C for 10 s, 50°C for 30 s, and 72°C for 30 s, and a final extension at 72°C for 5 min. Ultimately, sequencing libraries were generated using the TruSeq DNA PCR‐Free Sample Preparation Kit, and sequencing was carried out on an Illumina HiSeq2500 platform and 250 bp paired‐end reads in Novogene (Beijing, China).

### 2.4 Statistical analysis

Paired‐end reads were assigned to samples based on their unique barcode and were truncated by cutting off the barcode and primer sequence. We ensure a mismatch rate of no more than 0.1 and that the minimum PE reads were not lower than 10 bases in splicing. We partially intercepted the PE reads at the 3' end based on the length of the fragment and the length of the PE reads overlap, and we filtered out sequences with a continuous high‐quality base length of <75% of the length of the sequence length. Trimmed 16S microbial sequencing data were analyzed in QIIME (Quantitative Insights into Microbial Ecology, 1.9.1) (Caporaso et al., 2010). Microbial operational taxonomic units (OTUs) were generated using the clustering software UCLUST (Edgar, 2010) at an identity cutoff of 97%, which were compared with the Greengenes database. Singleton OTUs that did not match the reference database were removed while performing the analysis. The quality test was repeated. Clean reads were compared with SILVA_119_SSURef_Nr99_tax_silva.fasta to detect chimaeric sequences using USEARCH v7.0.1090, and then, the chimaeric sequences were excluded to obtain the effective tags for the final analysis. Finally, OTUs were rarefied by random sampling at an even depth of 53,349 reads to maximize the samples along with a complex downstream data analysis.

Alpha diversity analysis was performed using the Chao1, Good's_coverage, Observed_species, PD_whole_tree, Shannon, and Simpson_reciprocal indices. Data were expressed as the mean ± standard deviation (*SD*) and were analyzed from rarefied samples using QIIME. Statistical analysis was performed using SPSS 19.0 software, and the values are expressed as the mean ± *SD* (criterion of significance: *p* < 0.05). Beta diversity included both the unweighted and weighted UniFrac distances methods. Principal coordinate analysis (PCoA) was visualized using the unweighted UniFrac. Adonis function analysis was performed in R of vegan package and based on unweighted UniFrac. The relative abundances of the phylum levels were plotted as pie and bar graphs. A hierarchical clustered heatmap (Cluster 3.0 and Java Treeview) and bar graphs were used to reveal the relative abundance of genera. Linear discriminant analysis effect size (LEfSe) (Segata et al., 2011) was used to analyze the differences between the microbiomes of the GIT segments at the genus level. A Venn diagram was generated to show shared OTUs (http://jvenn.toulouse.inra.fr/app/example.html). The microbial function was predicted using PICRUSt (Langille et al., 2013) and STAMP software (Parks, Tyson, Hugenholtz, & Beiko, 2014). Welch's *t* test was used for data analysis, and a *p*‐value <0.05 was considered statistically significant.

## 3 RESULTS

### 3.1 Sequencing quality

We performed amplicon sequencing of the hypervariable V4 region of the 16S rRNA gene from a total of 15 gut tissue samples collected from the three snakes living in the wild*.* The median amplicon length was 253 bp after merging. After filtering, 1,175,594 high‐quality sequences were acquired from 1,278,028 raw reads, with an average of 78,373 reads per sample. These sequences resulted in a total of 3,666 OTUs. Each sample had an average of 1,170 OTUs. The sequences were assigned to 47 phyla, 124 classes, 233 orders, 407 families, and 746 genera.

### 3.2 Alpha diversity index analysis

Good's_coverage ranged from 99.01% to 99.3%, indicating that a sufficient number of 16S rRNA gene sequences were retrieved from the *R. subminiatus *gut segments to assess the maximum level of bacterial diversity. The results showed that the microbial abundance was significantly higher in the upper GIT segment (esophagus, stomach) and small intestine than in the lower GIT segment (large intestine, cloaca). In particular, the average Chao1 index and observed species value in the esophagus were significantly (*p* < 0.05) higher than those of the large intestine (Table 1). However, Shannon and Simpson_reciprocal diversity indices were found to be similar among all GIT tissue samples. Notably, we found that the small intestine had the highest diversity, while the large intestine had the lowest (Table 1 and Appendix A2). Furthermore, the average value of PD_whole_tree index was significantly (*p* < 0.05) higher in the esophagus (129.5678) than in the large intestine (88.9248) and cloaca (104.2179) (Table 1). The phylogenetic analysis revealed that the esophagus and stomach have the most similar microbial relationship, whereas the large intestine and cloaca are closer to each other.

**Table 1:** Statistical analyses of alpha diversity

	Chao1	Observed_species	Shannon	Simpson_reciprocal	PD_whole_tree	Good's_coverage
ES	1,846.6934 ± 207.3374^a^	1,219.7 ± 172.3552^a^	4.4334 ± 0.4734^a^	5.4012 ± 2.1931^a^	129.5678 ± 8.7688^a^	0.9901 ± 0.0009^c^
ST	1,693.5637 ± 95.1667^ab^	1,084.5667 ± 105.6584^a^	4.0325 ± 0.7075^a^	4.6827 ± 2.3689^a^	123.8604 ± 11.4742^ab^	0.991 ± 0.0006^bc^
SI	1,561.9169 ± 123.0370^ab^	1,071.7333 ± 318.9978^a^	5.166 ± 2.3238^a^	20.0021 ± 28.2141^a^	115.0529 ± 18.0191^ab^	0.993 ± 0.0013^a^
LI	1,313.2661 ± 28.3708^b^	722.7667 ± 67.3692^b^	3.389 ± 0.5194^a^	4.1697 ± 1.1089^a^	88.9248 ± 4.9962^c^	0.993 ± 0.0004^a^
C	1,381.6659 ± 252.0194^b^	892.2333 ± 163.9705^ab^	4.5815 ± 1.0546^a^	9.8711 ± 5.9512^a^	104.2179 ± 16.6447^bc^	0.9927 ± 0.0014^ab^

Note

Alpha diversity indices show the abundance and diversity of bacteria. Good's_coverage reflects the depth of sample sequencing, Chao1, and Observed_species reflects the bacterial abundance of samples, Shannon and Simpson_reciprocal reflect the diversity of samples, and PD_whole tree reflects the phylogenetic relationship of samples in the community. Different GIT segments were assigned as ES (esophagus), ST (stomach), SI (small intestine), LI (large intestine) and C (cloaca). Data are expressed as the mean ± *SD* (*n* = 3) with different letters in superscript representing no significance with other data with the same letter and a significant difference in the data with different letters (*p* < 0.05).

### 3.3 Composition and structure of the gut microbiota at different taxonomical levels

**Figure 1:**
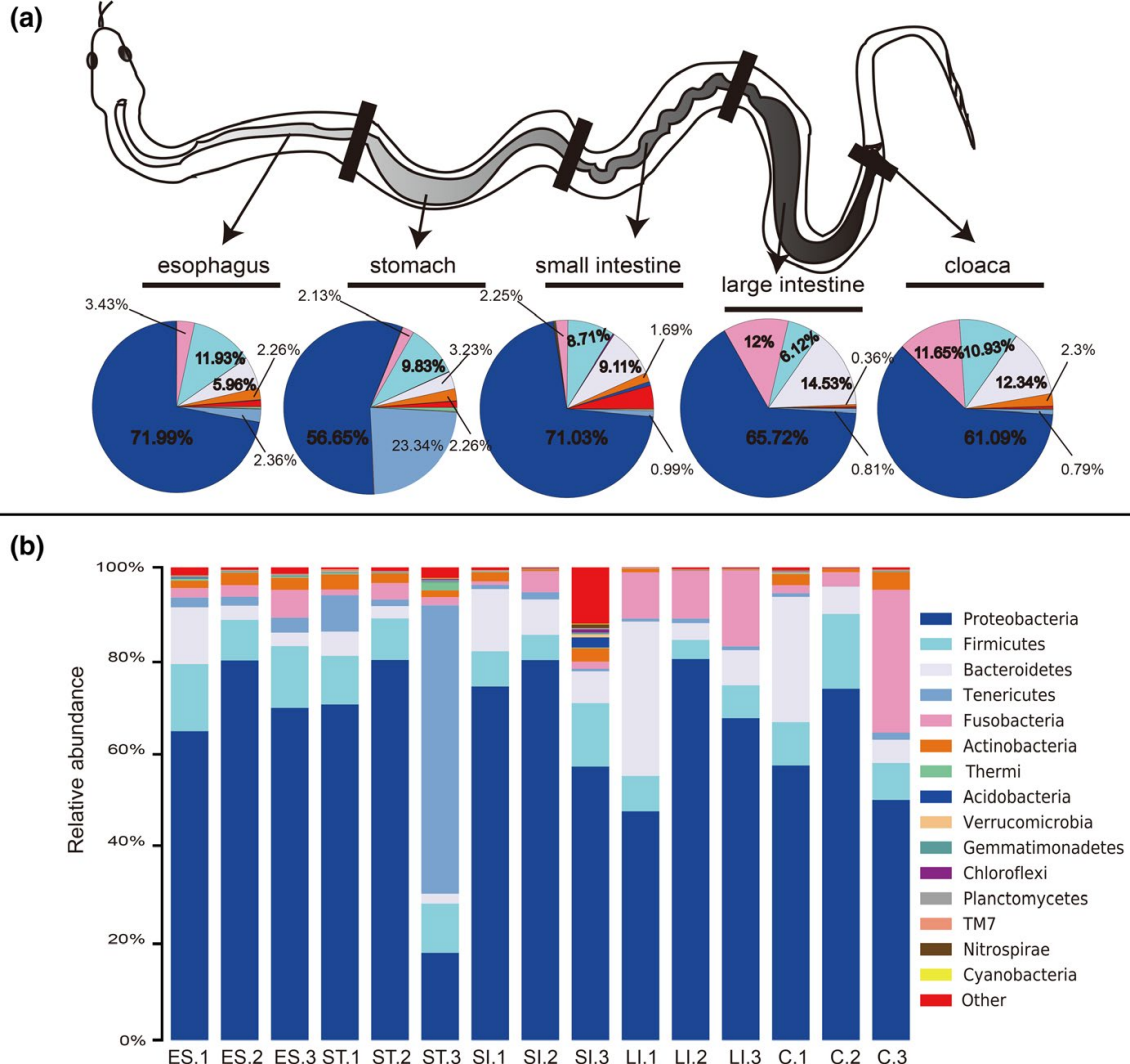
Relative abundance of gut microbiota composition in different GIT segments and individual samples at the phyla level. Gut microbiota composition in different GIT segments (a) and individual samples (b). The top 16 abundant taxa are shown with a pie and bar chart

The bacterial taxa contributed to separate microbial communities, and their relative abundance is described in Appendix A3. The most common taxa principally determined which belongs to Proteobacteria (65.30%), Firmicutes (9.5%), Bacteroidetes (9.03%), Fusobacteria (6.29%), Tenericutes (5.66%) as top five phyla. Among these, Proteobacteria was the most abundant phylum among five examined GIT segments. Afterward, the Firmicutes was the second most abundant in the esophagus, Tenericutes in the stomach, and Bacteroidetes was the secondary phylum in the small intestine, large intestine, and the cloaca. Subsequently, Bacteroidetes was the third most abundant phylum in the esophagus, Firmicutes was the third most abundant phylum in the stomach and the small intestine, and Fusobacteria was the third most abundant phylum in the large intestine and the cloaca. The small intestine harbored the maximum number of phyla (40 phyla), while the lowest number of phyla (32 phyla) was observed in the large intestine. Moreover, an obvious dynamic distribution of Bacteroidetes and Fusobacteria was found mainly in the lower GIT. Conversely, the distribution of Tenericutes was mainly in the upper GIT. We also found that the abundance of Actinobacteria in the esophagus and stomach was significantly higher than that in the large intestine (Figure 1a and Appendix A4). In addition, Proteobacteria was the predominant phylum in all tissue samples examined except ST.3, in which the Tenericutes phylum was the most abundant. We also found that SI.3 contained many other bacterial populations at the phylum level (Figure 1b). By combining the Shannon and Simpson_reciprocal indices, although the values were relatively high, we found that there was no significant difference. The gut microbiota diversity in SI.3 was relatively high compared to that of SI.1 and SI.2. Bacteroidetes was also prevalent in RS.1, especially in the lower GIT segments, and there was an obvious individual difference.

**Figure 2:**
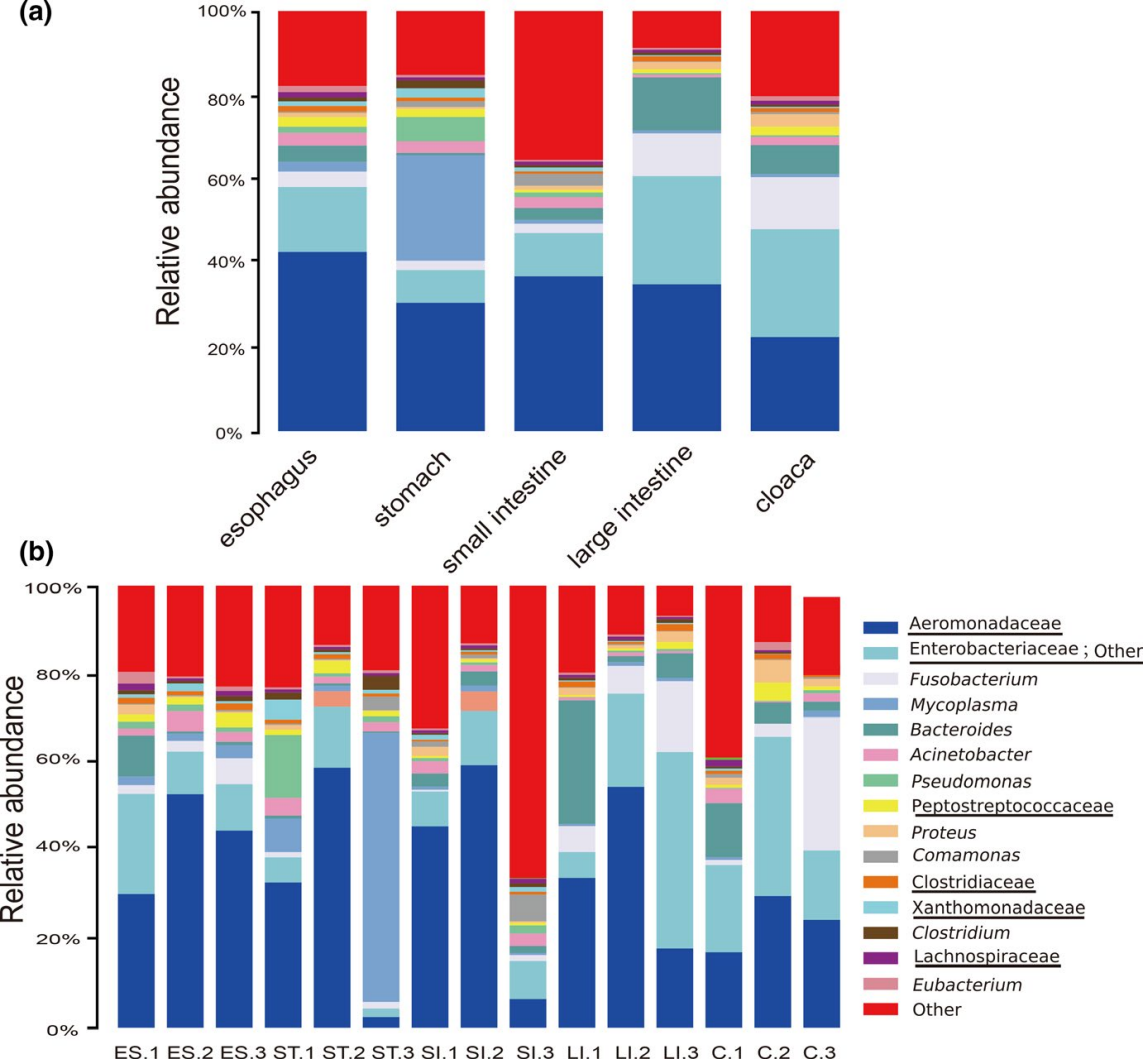
Relative abundance of microbial composition in different GIT segments and individual samples at the genus level. Gut microbiota composition in different GIT segments (a) and individual samples (b). The top 16 abundant taxa are shown with a bar chart (An underlined representative was classified only to the family level and the genus name was not accurately defined)

At the genus level, 747 bacterial taxa were detected in all GIT segments of *R. subminiatus*, but 50.33% of all sequences were not identified. The most prevalent genera in the GITs included *Fusobacterium* (5.67%), *Mycoplasma* (5.52%), *Bacteroides* (4.91%), *Acinetobacter* (2.03%) and *Pseudomonas* (1.71%), as well as unclassified genera belonging to the families Aeromonadaceae (34.09%), Enterobacteriaceae, other (15.73%), Peptostreptococcaceae (1.45%), Clostridiaceae (0.93%), and Xanthomonadaceae (0.88%) (Appendix A5). We found that Aeromonadaceae and Enterobacteriaceae were highly abundant in all GIT segments. *Mycoplasma* was mainly distributed in the esophagus and stomach. However, *Fusobacterium* and *Bacteroides* were more prevalent in the large intestine and cloaca than in the other GIT segments (Figure 2a). The overall microbiota compositions for each individual sample at the genus level showed that *Mycoplasma* was the predominant genus in ST.3, and SI.3 contained many other bacteria at the genus level (Figure 2b). Such differences were shown in the alpha diversity index and the phylum level.

**Figure 3:**
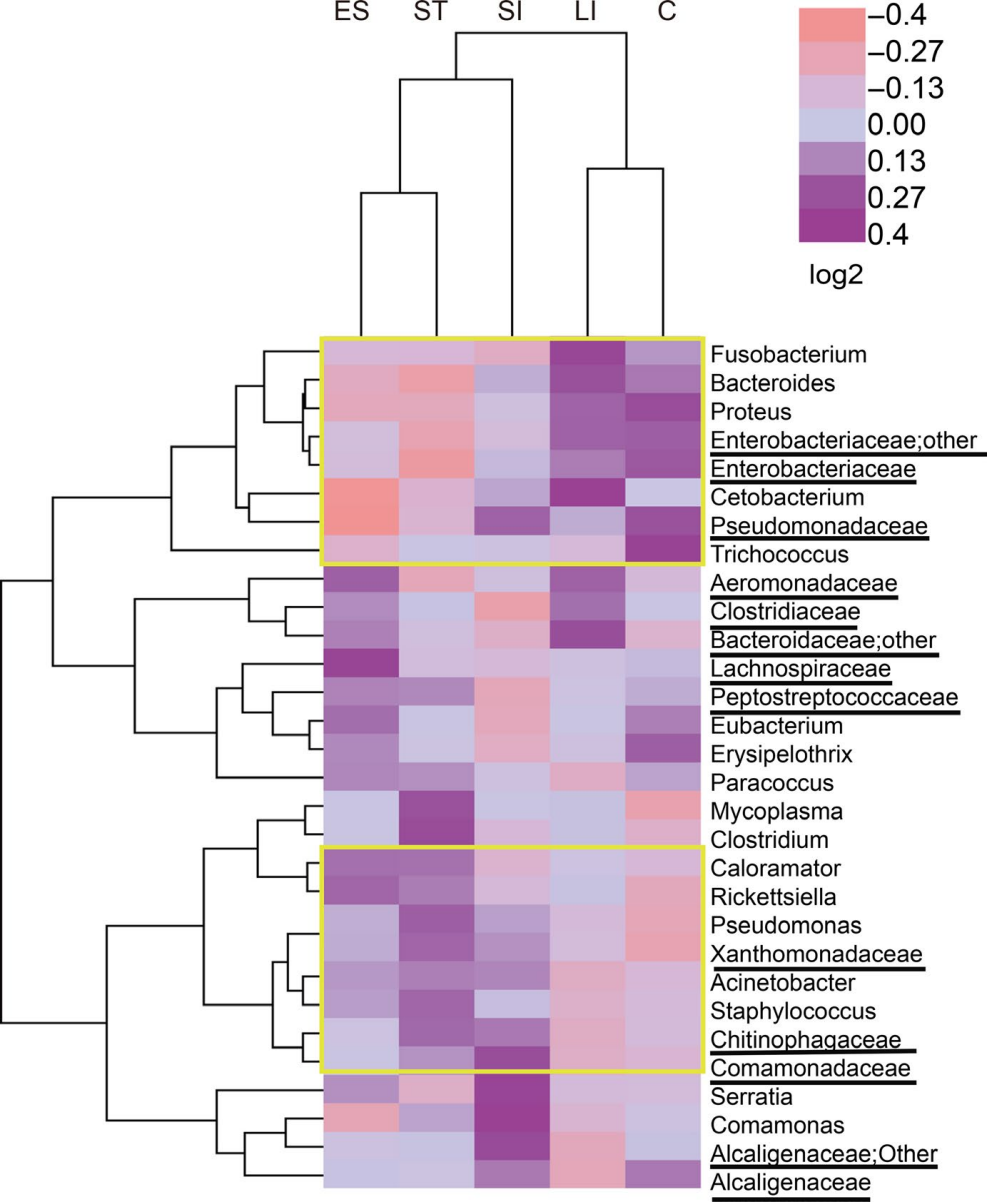
Heatmap of hierarchy cluster results for the abundance of the top 30 genera in different GIT segments (An underlined representative was classified only to the family level and the genus name was not accurately defined, “Other” representative when denoting classification, the program cannot judge which category should be classified according to the rules)

The hierarchy cluster heatmap of the top 30 genera highlighted the particularly high or low genera in the different GIT segments using a yellow frame. The results were similar to those in the bar chart. We found that the esophagus and stomach had many similar color modules on the abundance of the expression of genera. The large intestine and cloaca showed many similar color modules on the abundance of the expression of genera (Figure 3). We found that *Pseudomonas* was significantly more abundant in the esophagus than in the large intestine and cloaca, and *Rickettsiella* was more abundant in the stomach than in the small intestine, large intestine, and cloaca (Appendices A4 and A6).

**Figure 4:**
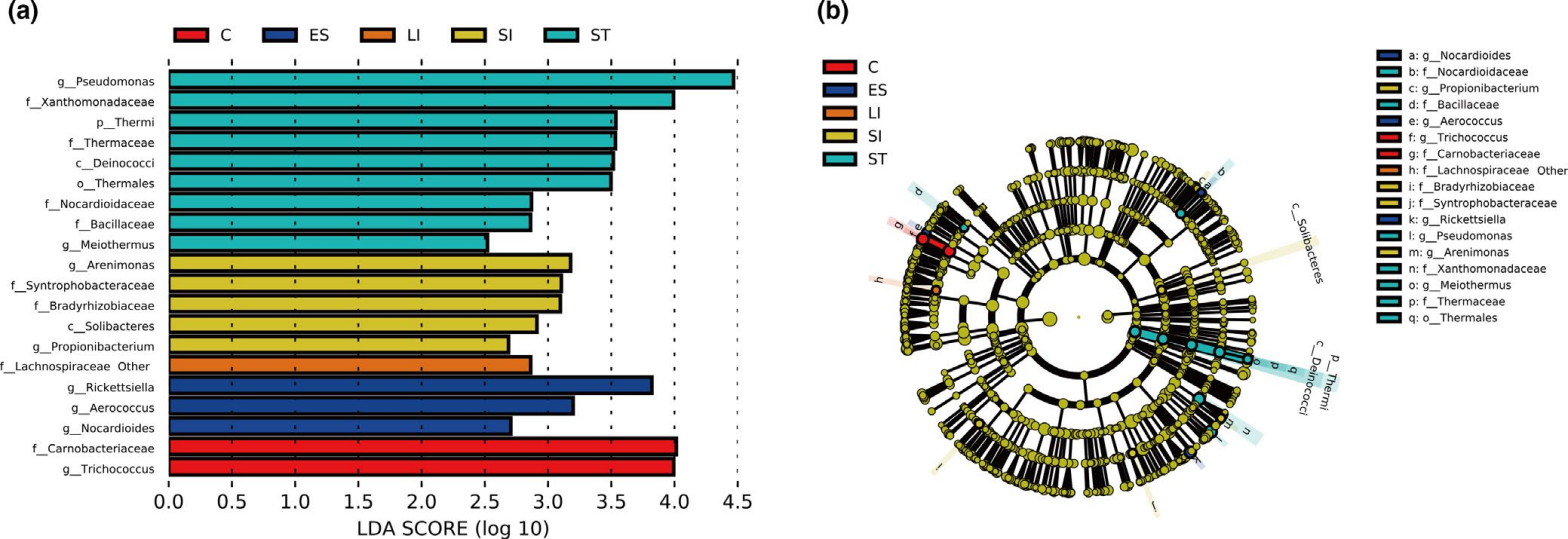
Linear discriminant analysis effect size (LEfSe) analysis of bacterial taxa was significantly different in the different GIT segments of *R. subminiatus* by the default parameters. A histogram of the LDA scores that were computed highlights different abundance among different GIT segments (a) (histograms of different colors represent the most significant differences in different GIT segments, abundance annotation represents phylum, class, order, family, and genus). Cladogram of bacterial taxa that were differentially abundant in different GIT segments (b)

We performed LEfSe analysis on all bacterial taxa to identify which were significantly different between GIT segments. The results showed that a total of 20 distinct bacterial taxa were found in the five intestinal segments. Three bacterial taxa were significantly abundant in the esophagus (e.g., *Rickettsiella* and *Aerococcus*), nine bacterial taxa were significantly abundant in the stomach (e.g., *Pseudomonas* and *Xanthomonadaceae*), five bacterial taxa were significantly abundant in the small intestine (e.g., *Arenimonas* and Syntrophobacteraceae), one bacterial taxa was significantly abundant in the large intestine (e.g., Lachnospiraceae; other), and two bacterial taxa were significantly abundant in the cloaca (e.g., Carnobacteriaceae and *Trichococcus*) (Figure 4a). In the cladogram, the stomach and the small intestine are closer together, whereas the large intestine and the cloaca are closer to each other. In addition, there was an abnormal value in the esophagus that crossed the cloaca (Figure 4b). These findings were similar to the results of alpha diversity and the heatmaps. Interestingly, there were significant differences observed in species abundance and diversity between the upper and lower GIT segments.

### 3.4 Relationship of the microbiota between the different GIT segments

**Figure 5:**
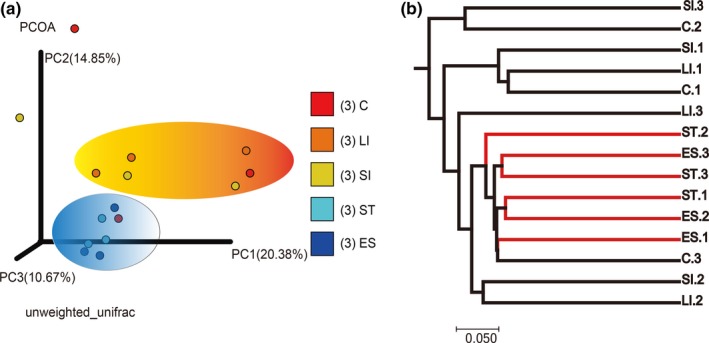
Differences in bacterial community structures and relationship between five GIT segments with unweighted UniFrac distances. Principal coordinate analysis (PCoA) of bacterial community structures of the gut microbiota in the five GIT Segments (a). Each solid circle symbol represented each gut microbiota and shows distinct bacterial communities between different GIT segments. The UPGMA tree analysis of five GIT segments through evolution (b)

The relationships between the microbiota structures of *R. subminiatus* were examined across different GIT segments using PCoA. The first component, PC1 (20.38%), separated the small intestine, large intestine, and cloaca of the RS.1 samples from the others (Adonis: *R* ^2^ = 0.188, *p = *0.004). The second component, PC2 (14.85%), separated the esophagus and stomach from the small intestine, large intestine, and cloaca (Adonis: *R* ^2^ = 0.122, *p = *0.017). The results show that the microbiotas of the esophagus and stomach were distinct from those of the small intestine, large intestine, and cloaca. However, no significant differences in community structure were observed between the samples of the esophagus and the stomach (Figure 5a). The UPGMA tree showed that the esophagus and stomach of *R. subminiatus* are similar in the way they have evolved (Figure 5b).

**Figure 6:**
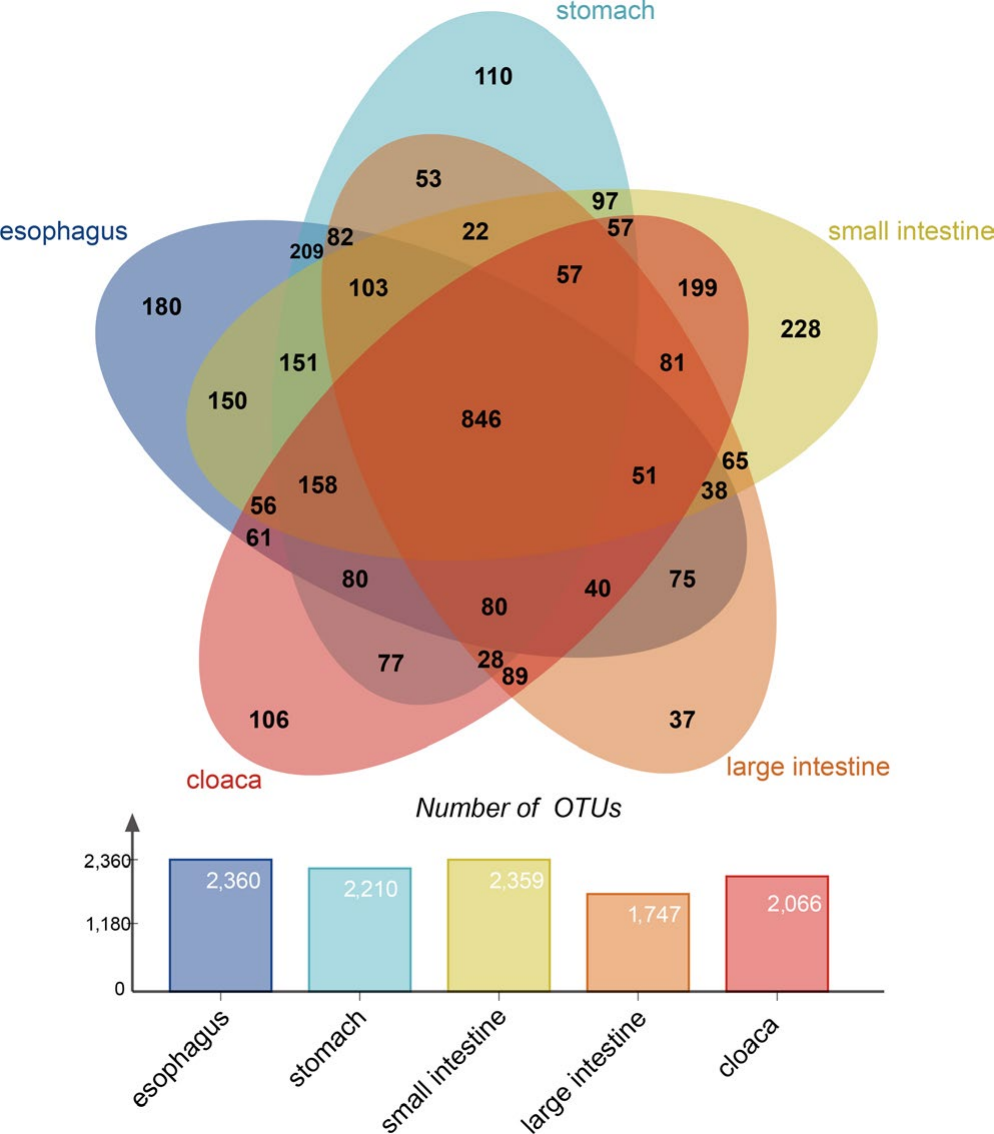
The OTU numbers of different GIT segments for the Venn diagram (The overlap regions show the common OTU numbers among different GIT segments)

A total of 3,666 OTUs were identified in all groups, and all GIT segments shared 846 OTUs. The esophagus, stomach, small intestine, and cloaca shared 158 OTUs; the esophagus, small intestine, large intestine, and cloaca shared 51 OTUs; the esophagus, stomach, and small intestine shared 151 OTUs; the stomach, large intestine, and cloaca shared 28 OTUs; the esophagus and stomach shared 209 OTUs; and the stomach and large intestine shared 53 OTUs. This finding indicated that the esophagus and stomach were more similar in terms of OTUs of gut microbe quantity compared with the large intestine and cloaca, which had low similarity (Figure 6). From the point of view of the OTU correlation coefficient, we found that the correlation coefficient in different GIT segments of the snake was still relatively high. The correlation coefficient between the stomach and the lower GIT segments (the large intestine and cloaca) was lower than that of the other GIT segments (Appendix S7). Interestingly, similar results were also obtained in the LEfSe analysis.

### 3.5 Predicted microbial function between the different GIT segments

**Figure 7:**
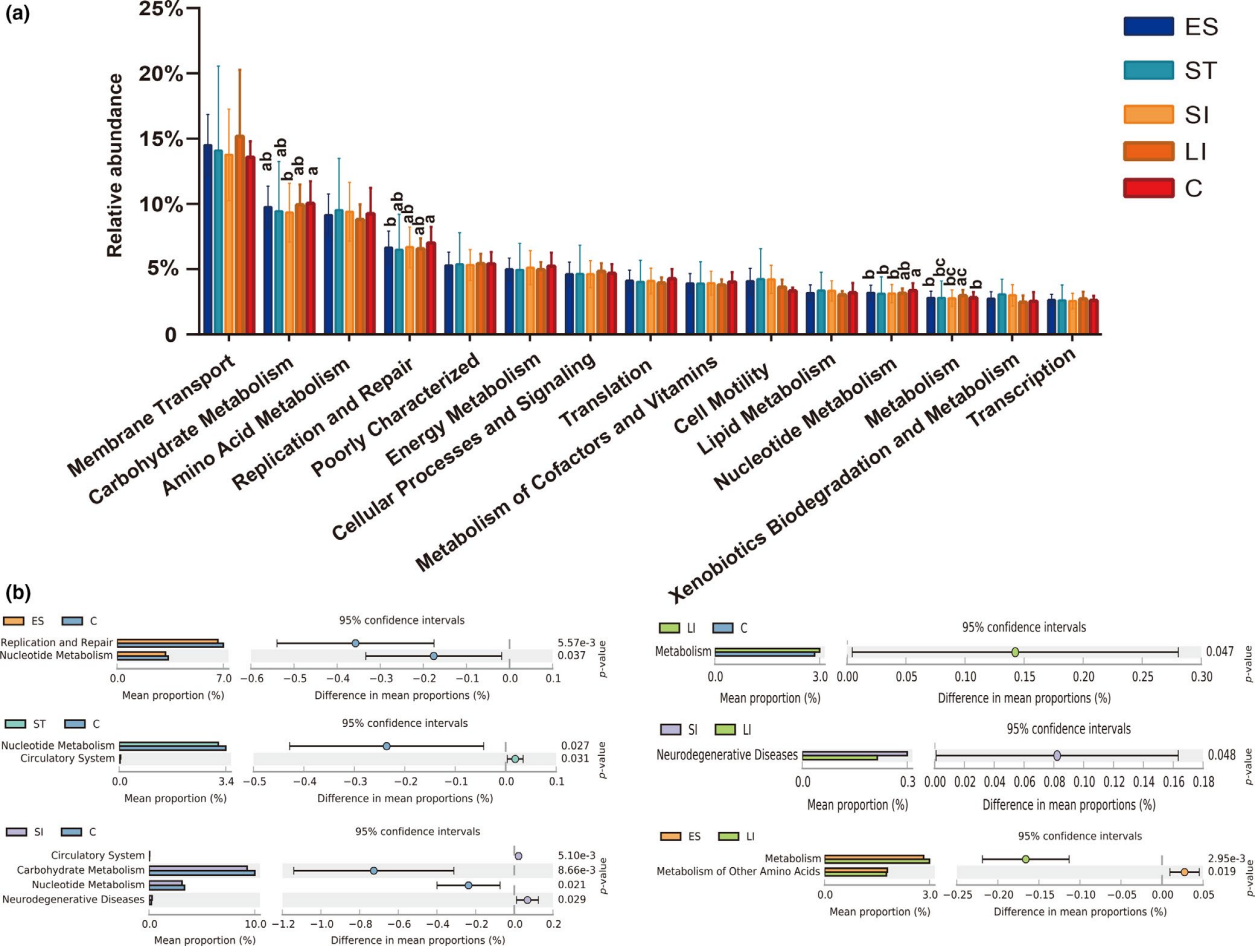
Microbial functional differences in different GIT segments. The relative proportions of the most abundant metabolism‐related KEGG pathways (level 2) predicted by PICRUSt between similar GIT segments of the top 15 (a). The error bars are standard deviations. The star indicates (*p* < 0.05) using Welch's *t* test (There was no significant difference in the content denoted with the same letter, but there was a significant difference in content denoted with the different letter). Comparison of microbial functions significant differences in different GIT segments (b)

Comparing the predicted microbial functions between the different GIT segments, we focused on the top 15 most abundant microbial function pathways. Gene function level 2 was enriched in the metabolic functions, in which eight of the top 15 functional pathways were categorized as related to metabolism. These include carbohydrate metabolism, amino acid metabolism, energy metabolism, metabolism of cofactors and vitamins, lipid metabolism, nucleotide metabolism, xenobiotic biodegradation, and metabolism. In addition, we also found that membrane transport, replication, and repair; cellular processes and signaling; translation; cell motility; and transcription were the main functional pathways of the snake gut microbiota (Figure 7a). There was no significant difference observed in microbial function between the esophagus, stomach, and small intestine. The large intestine had functional pathways that were significantly different from those of the esophagus and small intestine. Functional pathways related to the microbiota of the cloaca differed significantly from those of all other segments (Figure 7b). The circulatory system was mainly found in the stomach and small intestine. The major functional pathway associated with neurodegenerative diseases was found in the small intestine. Carbohydrate metabolism and metabolism were mainly found in the large intestine and cloaca (Figure 7a,b). Overall, there were significant differences in obvious microbial functions between the upper and lower GIT segments.

## 4 DISCUSSION

In reptiles, the dominant bacterial phyla are Firmicutes, Proteobacteria, Bacteroidetes, Fusobacteria, and Actinobacteria, but the proportion of each phylum is dynamic and is affected by multiple factors, such as animal species, gut morphology, and age (Ellegaard & Engel, 2016). In this study, we analyzed the bacterial diversity and abundance of *R. subminiatus*, a species with a broad geographic distribution species. We analyzed the microbiota across different GIT segments, and the individual differences in microbiota composition were discussed. The results showed a significant difference between the upper GIT segments (the esophagus and stomach) and the lower GIT segments (the large intestine and cloaca); the upper GIT segments showed a higher bacterial abundance than did the lower GIT segments (the large intestine and cloaca). Interestingly, the small intestine connects the upper and lower GIT segments and has common bacterial diversity. In contrast, in a study of different intestinal regions of carnivorous alligators, the oral cavity contained the most abundant diversity, with low abundance and diversity in the upper GIT segments and relatively high abundance and diversity found in the lower GIT segments (Keenan et al., 2013). Additionally, in omnivorous mice studies, the lower GIT segments contained high levels of bacterial abundance and diversity (Suzuki & Nachman, 2016). Although we did not take oral samples from the snakes, the upper GIT segments had higher abundance than did the lower GIT segments, but there was no significant difference in the diversity of the GIT segments. These results are consistent with an herbivorous bactrian camel study in which high abundance and diversity were found in the lower GIT segments (He et al., 2018), which is associated with the camels retaining feed particles in their rumen for much longer than other large herbivores do. It is known that snakes are carnivores and will eat their prey whole. This includes the fur, feathers, and bones of their prey and the undigested food in their prey's intestines (Holmberg et al., 2002). We assumed that different GIT segments of the snake are highly specialized compartments. We have sufficient reason to believe that symbiotic bacteria that are more diverse in the esophagus, stomach, and small intestine of snakes play an important role in digestion and absorption.

The composition of gut microflora in *R. subminiatus* plays an important role in digestion and absorption. At the phylum level, Proteobacteria was the predominant phylum in all GIT segments. Proteobacteria are facultative anaerobes and have been found to be the dominant phylum in the gastrointestinal tract of some fish (Givens, Ransom, Bano, & Hollibaugh, 2015), snakes (Colston et al., 2015), and birds (Xie et al., 2016). These bacteria typically breakdown and ferment complex sugars, and *Escherichia* may be important in the production of vitamins for the host (Colston & Jackson, 2016). In addition, a chronic prevalence of Proteobacteria in the gut can represent an imbalanced and unstable microbial community structure or a state of disease of the host. In a healthy intestine, Proteobacteria, also known as the commensal microbiota, have a protective role in immune responses against infection or inflammation (Shin, Whon, & Bae, 2015).

The gut microflora composition of *R. subminiatus* is similar to that of the wild timber rattlesnake (Mclaughlin et al., 2015). The most predominant phyla were Proteobacteria and Firmicutes in the stomach, small intestine, and colon, and the main metabolic pathway was carbohydrate metabolism. While studying the microbiome of the wild cottonmouth snake, researchers found that members of the phylum Bacteroidetes are the dominant bacteria of the large intestine, while the Proteobacteria phylum was dominant in the samples of the small intestine and cloaca (Colston et al., 2015). We have evidence that Proteobacteria are prevalent in wild snakes. In contrast, the gut microbiome of captive burmese pythons is dominated by members of the bacterial phyla Bacteroidetes and Firmicutes. Moreover, it has been found that during fasting, the members of Bacteroidetes in the large intestine are dominant, while an overall increase in abundance and diversity of Firmicutes is seen during the digestive process (Costello, Gordon, Secor, & Knight, 2010). There was a significant difference in the proportion of gut microbes in our study. The gut microflora of wild individuals may be significantly different from that of domestic animals. In addition, we could speculate that this difference is caused by eating different prey, because Burmese pythons feed on larger prey species such as rodents, while wild *R. subminiatus *tends to favor amphibians and fish. We found that diet plays an important role in driving the formation of intestinal microbes.

Both Aeromonadaceae and Enterobacteriaceae belong to the Proteobacteria phylum and are prevalent in all GIT segments. Aeromonadaceae are strict aerobes or facultative anaerobes typically associated with aquatic environments, and they produce acid from a variety of carbohydrates. They are spread by food, humans, and animals that have come into contact with water (Esteve, 1995). Members of this genus may be opportunistic pathogens in humans and animals, in which they can cause a range of extraintestinal infections or diarrheal diseases. However, information on their metabolism and ecology is relatively scarce (Huys,^2014^). Furthermore, a wild cottonmouth snake study purported an interesting conclusion that the increased prevalence of Proteobacteria suggests a gut microbiome more similar to that of birds (Colston et al., 2015). In our study, we found that the main bacterial taxon was Aeromonadaceae and speculated that wild *R. subminiatus *are more likely to survive near aquatic environments. Moreover, *R. subminiatus* likes to eat animals living in or near water. Interestingly, Enterobacteriaceae have been implicated in bloodstream infections and in cholangitis, peritonitis, and other intra‐abdominal infections. Additionally, organisms such as *Salmonella* cause gastroenteritis and subsequently, in some patients, invasive infection. *Klebsiella pneumoniae* correlates with pneumonia and colitis (Garrett et al., 2010; Paterson, 2006). The carnivore‐like structure of *R. subminiatus *could be further summarized as the prevalence of lineages from the family Enterobacteriaceae, which is found to be prominent in fecal samples from grizzly bears (Schwab, Cristescu, Northrup, Stenhouse, & Gänzle, 2011) and the giant panda (Guo et al., 2018; Xue et al., 2015). Enterobacteriaceae are significantly more prevalent in European children than in children in rural Africa. The diets of European children are mainly characterized by high animal fat and high‐protein contents (Filippo et al., 2010). Notably, Bacteroides spp. and Enterobacteriaceae are mainly found in the wild crotaline snake and the cottonmouth (Colston et al., 2015). Hence, carnivorous animals have a habit of eating high‐fat and high‐protein diets. Similarly, *R. subminiatus *had a microbial lineage typical of carnivores.

Our results raise an interesting question: why are Fusobacteria prevalent in the lower GIT segments of *R. subminiatus*? The same phenomenon exists in the study of the lower GIT segments of alligators (Keenan et al., 2013). Fusobacteria are mostly anaerobic and gram‐negative bacilli that produce butyrate and provide many benefits to the host, such as providing a majority of the energy supply for gut cells (Bennett & Eley, 1993; von Engelhardt, Bartels, Kirschberger, Düttingdorf, & Busche, 1998). Moreover, Fusobacteria are more likely to be involved in amino acid metabolism than glycometabolism, revealing a potential effect on protein degradation, which has been reported in the microbiomes of vertebrates such as alligators (Keenan et al., 2013), vultures (Roggenbuck et al., 2015), and some warm water fish (Larsen, Mohammed, & Arias, 2014). A previous study speculated that Fusobacteria in the lower GI tract of alligators may occupy a functional role in digestive organ development and nutrient acquisition that precedes a similar ecological niche that is now occupied by Firmicutes and Bacteroidetes in mammals (Keenan et al., 2013). In addition, *Fusobacterium*, which is an obligate anaerobe that can effectively breakdown proteins, produce butyric acid and metabolize carbohydrates, dominated in Fusobacteria. Many studies have found that it is associated with cancer, and its prevalence in the human colon is similar to that of cancer cells, but it is not yet clear whether it is involved in the formation of tumors or just uses the tumor for its own growth (Kostic et al., 2012; Mccoy et al., 2013). In our study, Fusobacteria represented 9% of the microbiome of the lower GIT segments. In contrast, Fusobacteria have been observed in higher abundances from composite microbiomes of humans and plays a key role in biofilm development (Mira, Pushker, Legault, Moreira, & Rodríguez‐Valera, 2004). This divergent niche occupation shows that different animals have different requirements. Although we lack oral samples, humans begin to digest food through chewing, unlike snakes that digest food through swallowing. We can speculate that the differences in the eating patterns of animals reflect the different compositional proportions of microorganisms. However, the mechanism of Fusobacteria that leads to this difference requires in‐depth research and discussion.

Bacteroidetes are strictly anaerobic and have the ability to break down polysaccharides and improve the rate of nutrient utilization. They are also likely to aid in the development of the host's intestinal mucosa and immune system, making them important for both carnivorous and herbivorous diets (Colston & Jackson, 2016). *Bacteroides *belong to the Bacteroidetes and are a commensal organism in the large intestine and cloaca, where they promote digestion and increase the utilization of complex carbohydrates (Spence, Wells, & Smith, 2006). In lower GIT segments, the Bacteroidetes would be considered the most prevalent microbial group in the timber rattlesnake, cottonmouth snake, and alligator microbiomes. In addition, this study found that RS.1 samples contained more Bacteroidetes and were significantly different in distance between the individual's small intestine, large intestine, and cloaca compared to other individual sample tissues in an analysis of PCoA and UPGMA trees. By combining with the body mass index of RS.1 samples, we can speculate that the composition ratio of Bacteroidetes was closely related to the body mass index of the host, and the abundance of Bacteroidetes was higher in individuals with lower body mass indices. Bacteroidetes may be the main factor of this clustering difference. This speculation is in agreement with a previous study of the composition ratio of Bacteroidetes in obese and lean hosts in which more Bacteroidetes were found in lean hosts (Filippo et al., 2010; Hildebrandt et al., 2009). Intestinal microbial composition is also strongly associated with age, A recent comparison of the gut microbiomes of tadpoles and adults specimens, and they find little overlap between bacterial communities from two different periods (Vences et al., 2016). The bacterial community differences could be linked with dietary preferences and physiological adaptations to digest different food (Vences et al., 2016). The results of our study revealed that subadult RS.1’s bacterial communities in lower GIT segments were different from adult individuals, but the lack of samples for a deeper exploration.

Tenericutes are completely inverse and are mainly distributed in the esophagus and stomach. Tenericutes are characterized by a lack of cell wall and typically have a very small genome and physical size. Within the vertebrate GIT, members of the Tenericutes have been identified as important members of the gut communities of fish and juvenile amphibians, where they may aid in nutrient processing, particularly for detritivorous hosts (Colston & Jackson, 2016). They have also been found to be dominant members of the microbiomes of the stomach in the giant African snail (Pawar et al., 2012). *Mycoplasma *(Tenericutes) is dominant in the stomach and the commensal bacteria colonizing a wide range of humans, mammals, reptiles, fish, birds, arthropods, and plants. Under certain conditions, *Mycoplasma* species are pathogenic and cause diseases in the hosts. Previously, it has also been found in the snake's stomach, and it can be inferred that it has a special effect on the stomach (Razin, Yogev, & Naot, 1998). Further research is needed to confirm whether *Mycoplasma* species are pathogenic, and they carry out some specialized function in the snake GIT or not.

*Pseudomonas* is a genus of gram‐negative Gammaproteobacteria belonging to the family Pseudomonadaceae. In our study, it was mainly prevalent in the esophagus and stomach. *Pseudomonas* bacteria are capable of breaking down proteins rather than carbohydrate fermentation. Moreover, they are a common bacterial infection in wounds and can cause bacteremia (Fazeli, Havaei, Solgi, Shokri, & Motallebirad, 2013; Plotkowski, Saliba, Pereira, Cervante, & Bajolet‐Laudinat, 1994). *Rickettsiella* is also prevalent in the esophagus and stomach, which comprises intracellular bacterial pathogens of a wide range of arthropods (Leclerque & Kleespies, 2008). Natural hosts include insects, arachnids, and crustaceans (Cordaux et al., 2007). We hypothesized that the accumulation of microbes in the snake's esophagus and stomach was mainly related to the location of snake in the natural food chain. Most amphibians feed on arthropods. Snakes ingest these amphibians and are often exposed to the aquatic environment. It is not difficult to infer that this indirect food intake and ecological environment leads to the accumulation of specific bacteria in the gastrointestinal tract. An interesting finding of our study is that the ecological niches of animals could be ascertained through the study of their gut microbes.

The results from the microbial function prediction suggested that membrane transport, carbohydrate metabolism, amino acid metabolism, replication, and repair pathways are common microbial functions in *R. subminiatus*. Gene function level 2 was prevalent in metabolism functions, which was in agreement with previous studies on the timber rattlesnake (McLaughlin et al., 2015), mice (Suzuki & Nachman, 2016), pigs (Zhao et al., 2015), cattle (Mao, Zhang, Liu, & Zhu, 2015), and bactrian camels (He et al., 2018). Comparing the predicted microbial function between different GIT segments, we detected a circulatory system that mainly exists in the stomach and small intestine. The circulatory system helps to stabilize temperature, pH, fight diseases, and maintain homeostasis (Tucker, 1966). The stomach and small intestine are involved more in amino acid metabolism. Furthermore, the small intestine was also contained more disease‐related systems (neurodegenerative disease). The large intestine and cloaca are mainly responsible for carbohydrate metabolism and other metabolism. Our study also found that the large intestine carries out more membrane transport. The reason for this phenomenon is that, as in omnivorous pigs (Zhao et al., 2015), the herbivorous bactrian camel (He et al., 2018) and the carnivorous timber rattlesnake (Mclaughlin et al., 2015), the stomach and small intestine are related to digestion and absorption, while the large intestine and cloaca are mainly responsible for microbial fermentation, unlike in ruminant cattle (Mao et al., 2015) in which more microbial fermentation occurs in the forestomach. Overall, different intestinal morphologies and physiological and biochemical environments are more likely to affect metabolic functions than diets are. Consistent with the findings in baleen whales and the giant panda, the evolutionary process of diet and intestinal morphology is not a coincidence but rather is the development of nutrient absorption and energy metabolism that can still meet the organism's survival needs (Sanders et al., 2015; Xue et al., 2015).

## 5 CONCLUSION

Based on high‐throughput sequencing technology, this study is the first to demonstrate the structure and distribution of gut microflora in different GIT segments of *R. subminiatus*. The results of our study revealed that the core wild *R. subminiatus* gut microbiome is comprised of Proteobacteria. In addition, our study confirmed the spatial heterogeneity of the GIT segments (i.e., the difference in the vertical distribution of intestinal microbes). An obvious dynamic distribution of Fusobacteria and Bacteroidetes was observed, mainly in the lower GIT segments. Conversely, the distribution of Tenericutes mainly existed in the upper GIT. Another finding in this study was the host‐specific nature of microorganisms, which was closely related to the individual's body mass index, age, gender, and health condition. Through high‐throughput sequencing methods, the effect of these variables on the proportion of microorganisms and the function of microorganisms was clearly demonstrated. One of the clear disadvantages of this study was the lack of samples because of many unavoidable limitations in sampling in the field environment. On the other hand, the study of snake gut microorganisms was interesting from the point of view of experimental design and evolution. In addition, most of the research is on the vertical inheritance of microorganisms by viviparous animals. However, we need to study how microorganisms are transmitted in oviparous animals. Moreover, for the relationship between disease and the microbial composition, this study also revealed that snakes carry a large number of potentially pathogenic bacteria. The snake can be a very good model animal for the disease research. We also found that Aeromonadaceae are prevalent in the GIT segments of snakes. The reason for this phenomenon was that this snake likely inhabits aquatic environments or areas near aquatic environments and has a great relationship with amphibians and fish. Research on the fasting and feeding of gut microorganisms by burmese pythons has also provided us with a direction (Costello et al., 2010). By studying hibernating animals, we can explore the effects of changes in seasonal food richness on gut microflora composition. Hence, snakes can be studied as a model of energy budget research by paying more attention to the gut microflora composition of wild snakes, which is critical for comprehensively understanding their evolution and ecology and improving the conservation of these captivating animals.

## CONFLICT OF INTERESTS

The authors declare that they have no conflict of interest.

## AUTHORS CONTRIBUTION

Wenjiao Tang, Guangxiang Zhu, and Diyan Li conceived and designed the experiments and drafted the manuscript. Wenjiao Tang, Guangxiang Zhu, Qian Shi, and Shijun Yang participated in the sample collection and DNA extraction, and Wenjiao Tang and Tianyuan Ma participated in the data analysis. Shailendra Kumar Mishra, Anxiang Wen, Huailiang Xu, Qin Wang, Yanzhi Jian, Jiayun Wu, MengXie, and Yongfang Yao assisted with the experiments and provided advice on the manuscript content, and all authors read and approved the final manuscript.

## ETHICS STATEMENT

Snakes were collected and sacrificed in accordance with IACUC protocols approved by the Institutional Animal Care and Use Committee of the Sichuan Agricultural University under the permit number SKY‐S20160709. The euthanasia of the animals took their welfare into full account. This study did not involve any endangered animals.

## Data Availability

The raw sequences used in this study were deposited on the NCBI Sequence Read Archive, and the SRA submission data are SRR7653064‐SRR7653078.

## APPENDIX A1

### Sample information

**Table:**

NGS‐Sample ID	Article‐ tissue ID	GI location	Raw PE	Effective Tags	gender	Total length	Body mass	Age	Captured locality	Time	Article‐ SampleID
288ES	ES.1	esophagus	87,243	75325	**♀**	515 mm	0.015 kg	Subadult	Guangdong	2017.10.13	RS.1
288ST	ST.1	stomach	95,905	89358							
288S	SI.1	small intestine	56,783	53349							
288L	LI.1	large intestine	90,009	85239							
288C	C.1	cloaca	92,104	86370							
289ES	ES.2	esophagus	95,371	90256	♂	742 mm	0.095 kg	adult	Guangdong	2017.10.13	RS.2
289ST	ST.2	stomach	97,362	91679							
289S	SI.2	small intestine	92,365	87893							
289L	LI.2	large intestine	83,271	79011							
289C	C.2	cloaca	66,921	62032							
290ES	ES.3	esophagus	84,656	62816	♀	1075 mm	0.185 kg	adult	Guangdong	2017.10.13	RS.3
290ST	ST.3	stomach	81,167	75674							
290S	SI.3	small intestine	68,303	60379							
290L	LI.3	large intestine	94,072	89004							
290C	C.3	cloaca	92,496	87209							

*Note*. NGS‐Sample ID is the name of the Next‐generation sequencing.

## APPENDIX A2

### Variations in alpha diversity of *Rhabdophis subminiatus* gut microbiota in the different GIT segments shown with a box‐plot

*Note*. Chao1 index, Observed_species index, Good's_coverage index, Shannon index, Simpson_reciprocal index and PD_whole_tree index.

## APPENDIX A3

### Taxonomy of *Rhabdophis subminiatus* gut microbiota at the phylum level

**Table:**

Taxon (phylum)	Article‐tissue ID					Mean
	ES	ST	SI	LI	C	
Proteobacteria	0.719908263	0.566534292	0.710277028	0.657204139	0.610926395	0.652970023
Firmicutes	0.119290017	0.098265329	0.087110655	0.061225062	0.109282701	0.095034753
Bacteroidetes	0.059566877	0.032340796	0.091071413	0.145338066	0.123357922	0.090335015
Tenericutes	0.023618545	0.233410886	0.009929599	0.008148105	0.007900743	0.056601576
Fusobacteria	0.034303973	0.021290762	0.022454232	0.120002396	0.116520461	0.062914365
Actinobacteria	0.022629028	0.022557647	0.016925753	0.003581799	0.022992005	0.017737247
Thermi	0.003565425	0.007516301	0.001352721	0.00055624	0.001179042	0.002833946
Acidobacteria	0.001442513	0.001016391	0.007533341	0.000299727	0.000462852	0.002150965
Verrucomicrobia	0.001194569	0.001091342	0.002488485	0.000349499	0.000829154	0.00119061
Gemmatimonadetes	0.000750688	0.001267811	0.001493661	0.000210734	0.000290096	0.000802598
Chloroflexi	0.000407627	0.000803136	0.002394463	0.000180796	0.00057529	0.000872262
Planctomycetes	0.000269969	0.000269446	0.000899347	0.000077207	0.000183662	0.000339926
TM7	0.000539035	0.001210874	0.00041008	0.000156538	0.000756591	0.000614624
Nitrospirae	0.000360562	0.000410604	0.002815135	0.000164793	0.000641369	0.000878492
Cyanobacteria	0.000205372	0.000436191	0.000495917	0.0000272	0.000070727	0.000247081
Other	0.011947536	0.011578208	0.042348199	0.002477772	0.004030944	0.014476532

*Note*. Top 16 relative abundance taxa.

## APPENDIX A4

### Comparison of differences between microbes of different GIT segments in phyla and genera

*Note*. (A) Comparison of differences between microbes of different GIT segments in phyla. (B) Comparison of differences between microbes of different GIT segments in genera.

## APPENDIX A5

### Taxonomy of *Rhabdophis subminiatus* gut microbiota at the genus level

**Table:**

Taxon(genus)	Article‐ tissue ID					Mean
	ES	ST	SI	LI	C	
Aeromonadaceae	0.425889513	0.31395259	0.371772964	0.35498154	0.237929485	0.340905218
Enterobacteriaceae;Other	0.14295462	0.071555761	0.095874357	0.23785566	0.238409208	0.157329921
Fusobacterium	0.034083102	0.020701374	0.020642973	0.093879501	0.114167262	0.056694842
Mycoplasma	0.02161979	0.23211481	0.008317902	0.006943679	0.007084654	0.055216167
Bacteroides	0.035201196	0.005023905	0.025890205	0.116203906	0.063174385	0.049098719
Acinetobacter	0.027961426	0.025808289	0.023476145	0.006217314	0.018239438	0.020340522
Pseudomonas	0.013874621	0.054039518	0.010677709	0.003338392	0.003514537	0.017088955
Peptostreptococcaceae	0.021346831	0.017622708	0.005860624	0.00860199	0.01899911	0.014486253
Proteus	0.009098238	0.004646714	0.008930288	0.015795236	0.027732935	0.013240682
Comamonas	0.002736688	0.012267181	0.026467082	0.001492298	0.004650581	0.009522766
Clostridiaceae	0.012616147	0.008482887	0.00554371	0.01127501	0.008728128	0.009329176
Xanthomonadaceae	0.010226089	0.01950445	0.008543144	0.00253123	0.003021712	0.008765325
Clostridium	0.00887484	0.017899299	0.005424821	0.00594926	0.00473488	0.00857662
Lachnospiraceae	0.010940733	0.006498017	0.007912779	0.006203267	0.008855134	0.008081986
Eubacterium	0.013572532	0.005202748	0.003383443	0.003966973	0.00972456	0.007170051
Other	0.209003633	0.184679747	0.371281855	0.124764743	0.231033989	0.224152793

*Note*. Top 16 relative abundance taxa (An underlined representative was classified only to the family level and the genus name was not accurately defined).

## APPENDIX A6

### Heatmap for the abundance of the top 30 genera based on log2FPKM+1 in different GIT segments of *Rhabdophis subminiatus*

*Note*. There was no significant difference in same letter, whereas there was a significant difference in different letters, *P*< 0.05.

## APPENDIX A7

### Correlation among OTUs of gut microbes from different GIT segments

**Table:**

	ST(3)	SI(3)	LI(3)	C(3)
ES(3)	0.8917449	0.9932243	0.9177545	0.8540797
ST(3)		0.8796523	0.7769082	0.7408613
SI(3)			0.9006663	0.8369843
LI(3)				0.8452662

*Note*. Three gut segments from three individuals were used to calculate the correlations.
